# Silica Aerogel-Rubber Composite: A Sustainable Alternative for Buildings’ Thermal Insulation

**DOI:** 10.3390/molecules27207127

**Published:** 2022-10-21

**Authors:** Patrícia Alves, Diogo Azeiteiro Dias, Ana Dora Rodrigues Pontinha

**Affiliations:** University of Coimbra, Chemical Process Engineering and Forest Products Research Centre, Department of Chemical Engineering, Rua Sílvio Lima, 3030-790 Coimbra, Portugal

**Keywords:** silica aerogels, sol-gel, recycled tire rubber, thermal insulation buildings

## Abstract

Silica aerogel composites with recycled tire rubber have been synthesized and evaluated for their potential use for thermal protection in buildings. The present work describes for the first time the preparation of silica-based aerogel composites containing recycled rubber tires reinforced with polyvinyl butyral (PVB) by hot pressing. The developed composite was extensively characterized regarding its physical, morphological, thermal and mechanical features, and the results showed their properties were relevant, leading to composites with different properties/performances. The obtained bulk density values were satisfactory, down to 474 kg·m^−3^, and very good thermal properties were achieved, namely, thermal conductivity as low as 55 mW·m^−1^·K^−1^ for composites with silica aerogel, recycled tire rubber and PVB. The most promising composites were those based on low bulk density and thermal conductivity values, and they were thermally stable, indicating their suitability for thermal insulation applications.

## 1. Introduction

Silica-based aerogels are popular in terms of production volume and real-world applications [1]. They have many unique and favorable properties due to their nonporous structural nature [2]. Silica aerogels have received great attention due to some of their properties such as very high pore volume (>90%) and a usually mesoporous network, which provide a very high specific surface area (500–1200 m^2^ g^−1^) [3], low density (∼0.03–0.3 g·cm^−3^) [4], low thermal conductivity (12–25 m W·m^−1^·K^−1^), ultra-low dielectric constant (k = 1.0–2.0) and low refractive index (∼1.05) [5,6]. The very low thermal conductivity [2,7] of silica aerogels, associated with nonflammable characteristics, make them suitable materials for thermal insulation systems. However, they have limitations in their mechanical strength and are complex to process without causing fractures in the final material [8]. Different approaches have been explored to improve the mechanical properties of silica aerogels [9], such as structural reinforcement using flexible silica precursors in silica gel backbone [10,11], dispersing fibers in the initial sol of silica aerogel [12] and conformal coating of silica backbone by surface cross-linking with a polymer [13,14,15].

Due to their unusual physical and chemical features, aerogels have gained great interest, especially for energy-efficient retrofitting solutions for buildings [16]. Aerogels are a state-of-the-art thermal insulation solution with great potential at the moment [5,17,18,19,20]. A common passive strategy for the reduction in energy consumption is the incorporation of thermal insulation materials in buildings’ envelopes [8,21,22].

On the other hand, the use of discarded tires in the construction industry is an economical and sustainable solution to meet future environmental challenges. The disposal of waste tires to landfills creates serious environmental problems due to the leaching of harmful and toxic elements into the surrounding ecosystem.

In the last decade, there has been a growth in the number of tires discarded as end-of-life tires (ELTs) [23], which leads to serious environmental problems. Approximately 70–80% of the total mass of the tires is vulcanized rubber, and their disposal is an issue, as they are nonbiodegradable and cannot be reprocessed in a simple process such as the thermoplastic materials remaining in the landfills [23,24,25]. Recycled rubber often lacks the correct balance of disulfide bridges between polymer chains, which are normally formed during the vulcanization stage [26], and the rubber can enter an “overcure” phase, causing it to have a lower tensile strength [27]. In order to ameliorate this problem, polymers are often added.

Polyvinyl butyral (PVB) is an interesting polymer with outstanding mechanical properties and excellent optical clarity [28]. The static mechanical property of PVB has significant deformability and is sensitive to strain rate and temperature [29].

In this work, motivated by the environmental problems caused by ELTs, the goal was to develop a composite of aerogel, recycled tire rubber waste and PVB using the hot-pressing technique. A comparative study on the influence of the different components on the physicochemical, thermal and mechanical features of the resulting composites was carried out. Due to its low thermal conductivity and good mechanical resistance, the aerogel-based composite has the potential for thermal insulation applications.

## 2. Results and Discussion

Since the main goal of this study is to evaluate the influence of aerogel, recycled tire rubber and polymer on the properties of composites, several characterization techniques were employed.

### 2.1. Chemical Characterization

**Figure 1:**
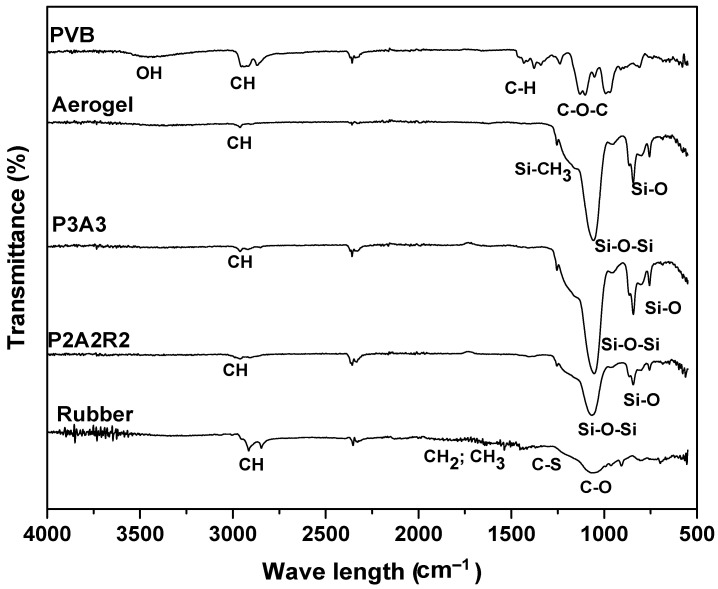
FTIR spectra for PVB, aerogel, rubber and different aerogel composites: P3A3 (PVB and aerogel composite) and P2A2R2 (PVB, aerogel and rubber composite).

The ATR-FTIR spectra of the different components of the composite, PVB, aerogel and rubber, and two different composites are presented in Figure 1.

All infrared spectra presented in Figure 1 show a series of bands in the 2980–2850 cm^−1^ range assigned to the C–H symmetric and antisymmetric stretching vibrations of CH_3_ and –CH_2_ groups [30]. These bands are more evident for PVB and rubber spectra due to their hydrophobicity. Other typical bands at 1377 cm^−1^ and 1463 cm^−1^ ascribed to –CH_3_ symmetric deformation vibrations and bending deformation are also present [31], especially in the PVB spectrum.

The O–H band observed at 3600 cm^−1^ in PVB is due to the predominance of hydroxyl groups (major), butyral and (less) acetyl groups in its composition [32]. In addition, a band at 1132 cm^−1^, which corresponds to the C–O–C butyral ring stretching is a typical band of PVB [31].

The rubber sample spectrum shows symmetric stretching of C–O–C and twisting of –CH_2_– at 1080 cm^−1^ and symmetric C–S–C group stretching vibrations in the two C–S bonds at 1250 cm^−1^. It is also possible to perceive the deformation of –CH_2_– and rocking of –CH_3_ and asymmetric deformation of –CH_3_ in the 1500–1700 cm^−1^ range [33,34].

For all the silica aerogels samples, it is possible to observe the stretching vibrations bands from Si–O–Si bonds of the SiO_2_ network: near 755 cm^−1^ (symmetric) and 1055 cm^−1^ (asymmetric), which are attributed to different modes of O–Si–O and Si–O vibration [35]. Regarding the –CH_3_ groups resulting from the sialylation, a band can be found at 2960 cm^−1^, corresponding to the symmetric stretching vibration of C–H from methyl groups.

Observing the spectra of composites P3A3 and P2A2R2, it can be seen that they are similar with bands at 3000 cm^−1^, 2300 cm^−1^ and 1000 cm^−1^, despite the fact that one of the composites has rubber in its composition (P2A2R2). The presence of rubber was not possible to be confirmed based on the FTIR spectra. Both spectra are more similar to the aerogel spectrum, which might be explained by the fact that the ATR-FTIR analysis only detects the composition at the surface of the composite and, since it is not homogeneous (is a mixture of the three components) it may be detecting a section with more aerogel than rubber or polymer. It should also be noted that, in the composite, many peaks overlap, and hence they cannot be properly identified.

### 2.2. Structural and Morphological Features

Thermogravimetry analysis (TGA) was performed to evaluate the thermal stability of the materials used since high temperatures were employed in the composites processing procedure (around 150 °C).

**Figure 2:**
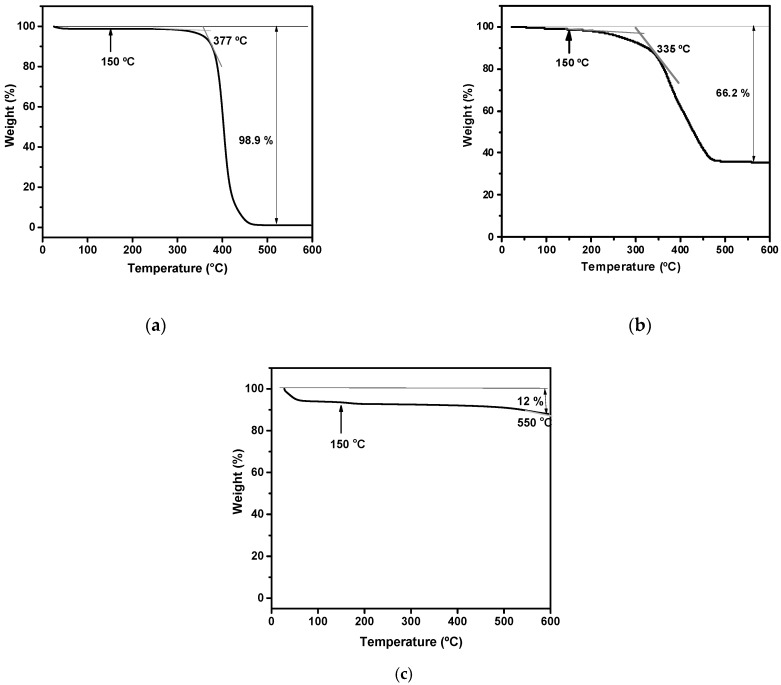
TGA traces of: (**a**) PVB; (**b**) rubber; (**c**) silica aerogel.

PVB TGA shown in Figure 2a displays a slight mass loss around 100 °C, resulting from the loss of residual water present in the sample. PVB showed good thermal stability, starting to degrade only at 377 °C, and presented only one step of degradation.

From Figure 2b, it is possible to observe that rubber presents good thermal stability, with an initial decomposition temperature of 331 °C. Until 250 °C, a weight loss of 4.3% was detected, which is probably due to the thermal degradation of the remaining plasticizer portions presented in the rubber [36].

Figure 2c shows a weight loss of silica aerogels near 60 °C. This is the result of the evaporation of entrapped H_2_O and alcoholic groups from hydrophilic silica aerogels as a result of the condensation reactions of Si–OH and Si(OC_2_H_5_) groups [37].

### 2.3. Surface Hydrophobicity

The water contact angle gives information about the surface hydrophilic/hydrophobic character, which strongly influences the interaction between the material and its surroundings (such as other materials or moisture). The results presented in Table 1 show the obtained values of the static water contact angle.

The results obtained confirmed the hydrophobic character of the composites, as well as their base materials (PVB and rubber). All composites showed higher water contact angles than rubber (R6) and PVB (P6); additionally, by analyzing the results, a tendency can be inferred: the higher the aerogel content in the composite, the more hydrophobic the composite is. This is related to the hydrophobic groups of the silica aerogel [38].

### 2.4. Density and Thermal Conductivity

**Figure 3:**
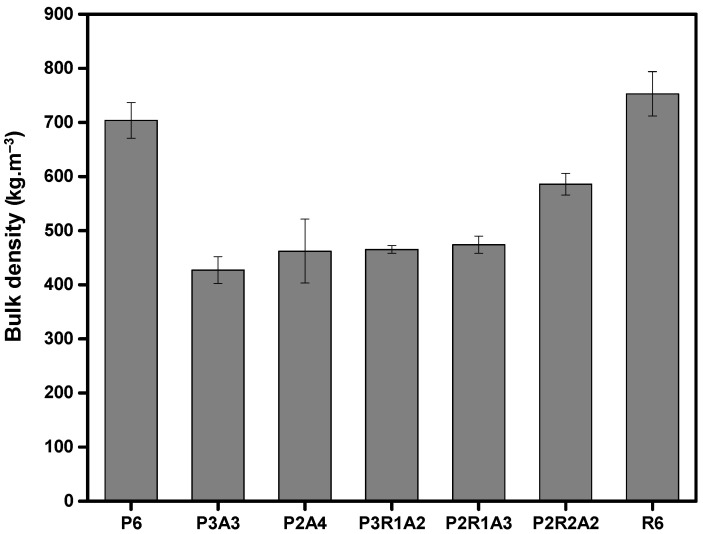
Bulk density for different samples.

The influence of the three different components, aerogel, PVB and rubber, on the bulk density was investigated, and the obtained results are given in Figure 3. The denser material, as can be seen in Figure 3, is recycled tire rubber, followed by PVB. However, the incorporation of aerogel into the composite led to a decrease in the bulk density. This result was expected due to the characteristic low bulk density of aerogels [39]. Therefore, and as expected, the addition and increase in rubber to the composite induced an increase in the bulk density of the composites.

**Figure 4:**
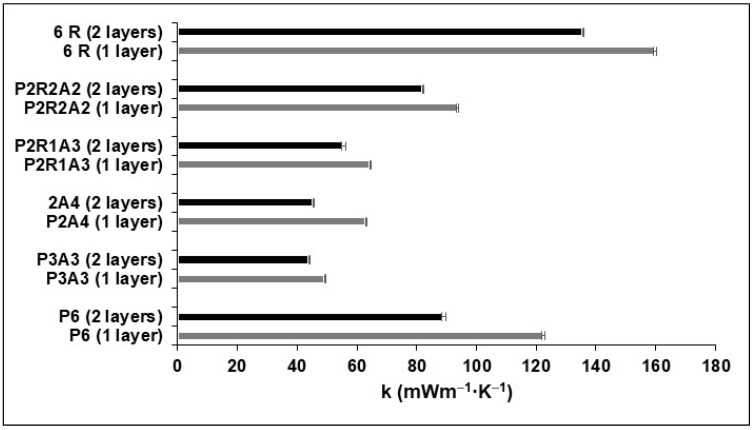
Thermal conductivity for single and double layers.

The thermal conductivity values tendency (Figure 4) agrees with the trend of the bulk density. Generally, both values decrease with aerogel addition due to the increase in porosity [40]. The composite with a lower thermal conductivity value was P3A3 (44 mW.m^−1^ K^−1^), which does not contain rubber. By adding 1 g of rubber and removing 1 g of PVB from the composite (P2R1A3), the thermal conductivity value remains relatively low, around 55 mW.m^−1^K^−1^, which makes it a great composite candidate for the insulation of buildings.

Since the results were promising for P3R1A2, the conductivity of samples with double the thickness was also measured (two samples of the same composite overlaid). The obtained results are also represented in Figure 4, in which a reduction in the conductivity value that varies between 10 and 30% from single to double layer can be observed for composites without rubber. This reduction was less significant for samples that include the three components: aerogel, PVB and rubber.

### 2.5. Mechanical Characterization

**Figure 5:**
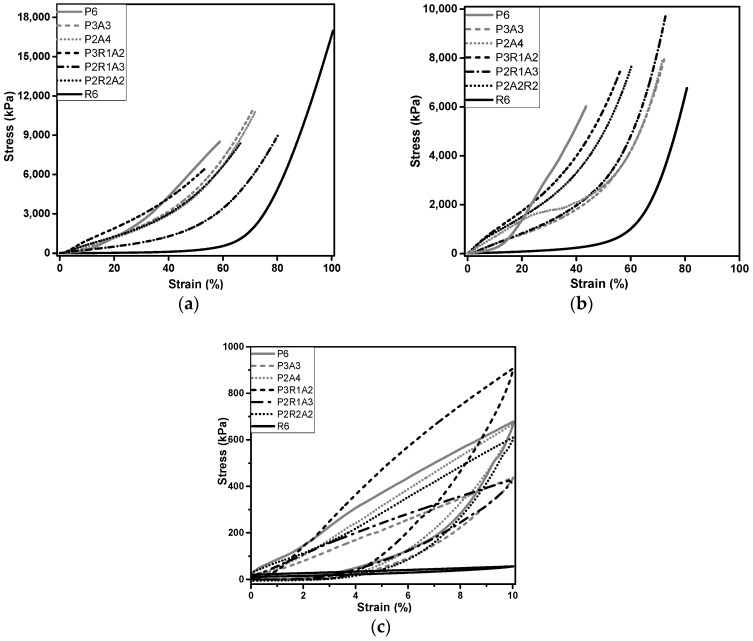
Stress–strain curves obtained in uniaxial compression mechanical tests with different compounds tested: up to maximum load of 3 kN for: (**a**) one-layer samples; (**b**) two layers samples; and tested (**c**) under a uniaxial compression–decompression cycle for one-layer samples.

The main purpose of this material is to be used in civil construction applications; therefore, mechanical properties are crucial, in particular the ability of the material to recover after compression. The mechanical behavior of the different samples was assessed by uniaxial compression tests under the same conditions up to the maximum load of the compression cell (2.8 kN) and are presented in Figure 5. Different values of maximum compressive strain were obtained for the tested materials as expected since, in the tests, the strain was not set at a chosen value. The different values observed are related to the different chemical structures of the different components, which can generate different mechanical resistance. Liu et al. [41] divided the mechanical behavior of PVB under quasi-static compression into three different stages similar to our results in Figure 5. In the first region, corresponding to a linear–elastic region, the stress of PVB increases gently along with the strain because of its large particle distances. An increase in stress is observed in the second region, which is likely due to the movement of dislocations [42,43]. In the final stage is present a significant increase in the slope of the stress–strain curve due to the densification of the porous structure, and consequently, the stiffness of the compressed sample increases.

The different values of maximum stress observed between one-layer and two-layer samples were possibly due to slippage between layers.

From the curves in Figure 5, it can be seen that rubber is the least stiff material.

The Young’s modulus was evaluated from the linear region of stress–strain loading curve of the compression test, and the results are shown in Table 2. The values obtained have different orders of magnitude among the samples, PVB being the one with the highest value and rubber the lowest value. The greater the Young’s modulus, the more rigid the material or the lesser the elastic deformation for the same applied load. All samples analyzed contained PVB with the exception of R6, which contains only rubber. The highest value was obtained for the sample with the higher amount of PVB, i.e., as expected. The introduction of aerogel leads to a decrease in Young’s modulus, as is the case of the P3A3 and P2A4 samples. By adding rubber, the value of the Young’s modulus decreases even more. The sample with the higher Young’s modulus is P2R2A2, making it the more compact and rigid sample.

Recovery tests were performed, and the results indicate that in general, after 10% strain, the samples restore up to 70–98% of their shape (Table 2). These results indicate an excellent mechanical performance of the composites in terms of flexibility. A good capacity of these materials to recover their original shape during unloading is very important considering the construction application.

### 2.6. Surface Morphology

**Figure 6:**
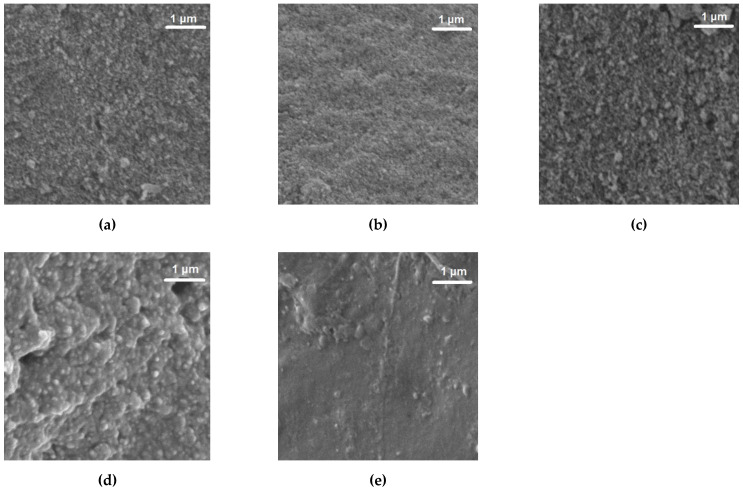
SEM images of materials: (**a**) P2R2A2, (**b**) P2R1A3, (**c**) P3A3 and samples (**d**) R6 and (**e**) P6.

The significant difference obtained in the Young’s modulus of the different composites is probably due to the different interaction between PVB, rubber and silica aerogel during processing. To better understand the morphology of the composites, SEM images were recorded and are displayed in Figure 6.

The images show that PVB acts as the binder of the composite since its presence improves the smoothness of the composite along with its homogenous appearance. On the opposite way, rubber sample (R6) shows several agglomerates, inducing an increase in the roughness of the composites with rubber; however, these roughness/agglomerates are more evident with the increase in silica aerogels content in the composites, which might be explained due to the silica aerogels’ properties, including the ability to increase porosity [44].

These results are in line with the results obtained from the mechanical tests: PVB is more compact, and it presented the highest value of Young’s modulus, rubber has a lower value of Young´s modulus, and in the SEM images, we can observe agglomerates.

## 3. Materials and Methods

### 3.1. Materials

For composite production were used the following materials: tetraethoxysilane (TEOS, purity ≥99%, Aldrich, Portugal, Si(OC_2_H_5_)_4_), peracetic acid (38–40%, Merck, Portugal, CH_3_CO_3_H), ethanol (absolute, Fluka, Portugal, C_2_H_5_OH), ammonium hydroxide (25% NH_3_ in H_2_O, Fluka Analytical, NH_4_OH), n-hexane (purity > 95%, Fisher Chemical, Portugal, C_6_H_14_,), hexamethyldisiloxane (HMDSO, purity > 98%, Acros Organics, Portugal, (CH_3_)_3_SiOSi(CH_3_)_3_,), trimethylchlorosilane (TMCS, purity ≥98%, Sigma Aldrich, Portugal, (CH_3_)_3_SiCl,), polyvinyl butyral (PVB, 60H, Kuraray America, Inc., C_14_H_24_O_5_) and tire rubber with a diameter <0.8 mm (provided by Amorim Cork Composites®, Mozelos, Portugal).

### 3.2. Synthesis of the Aerogel

The first step for the silica aerogel synthesis consisted of the addition of ethanol, TEOS and double-distilled water, with a molar ratio of 10:1:4, and then the mixture was stirred for 30 min. The silica gel was obtained by a low-temperature sol–gel process. The nanostructured solid network of silica is formed as a result of hydrolysis and condensation reactions of the silica precursors, in which siloxane bridges (Si–O–Si) are formed. The hydrolysis step involves the conversion of the alkoxide to silanol. The hydrolysis of silicon alkoxides was carried out with a catalyst [7]. A basic solution, NH_4_OH 2.5 M, was added to the former solution and kept under strong agitation for 1 min. The samples were kept in an oven at 27 °C for 5 days for aging.

The gels were cut into small sizes and washed with ethanol and hexane at 50 °C. The samples were then subjected to a surface modification procedure: sialylation. The sialylating solution comprises hexane, HMDSO and TMCS (70:20:10 v/v%). The gels were immersed in the sialylating solution and then placed in an oven at 50 °C for 6 h. To dry the gel, the solution was removed, and the gel was kept in the hood for 24 h and then at 100 °C for 3 h and 150 °C for 3 h.

### 3.3. Synthesis of the Aerogel–Rubber Composites

The synthesized aerogel, previously grounded, was mixed with recycled tire rubber and PVB (<0.8 mm), in different mass proportions. The mixture was placed into a metallic mold and was pressed (1.03 × 10^8^ Pa) in a laboratory hydraulic press (Carver Laboratory Press, Model C. 12 ton, heated 6’’ platens form Carver, Inc., Wabash, USA) for 5 min at 150 °C. The different composites produced (41 mm × 41 mm × 6 mm) are shown in Table 3.

### 3.4. Characterization of the Composites

The properties of aerogel, PVB, tire rubber and final composites were assessed by different characterization techniques. The chemical structure was evaluated by attenuated total reflection (ATR) Fourier-transform infrared spectroscopy (FTIR) (FT/IR 4200, Jasco, Tokyo, Japan) (ATR-FTIR), collecting the spectra between a wavenumber of 4000 and 400 cm^−1^, with 128 scans and 4 cm^−1^ of resolution. The bulk density (*ρ*_b_) was determined from the weight and volume of regular pieces of the samples. The degree of hydrophobicity was determined through contact angle measurements by an OCA 20 system (Dataphysics, Filderstadt, Germany) at room temperature using the sessile drop method and high-purity water. Scanning electron microscopy (SEM) images were obtained using a Compact/VPCompact FESEM (Zeiss Merlin, Leipzig, Germany) microscope after coating the aerogel samples with a thin gold layer by physical vapor deposition for 20 s. Thermal properties were assessed by thermal gravimetric analysis (TGA) and thermal conductivity. The thermal stability of different materials was obtained by using DSC/TGA equipment (TGA-Q500, TA Instruments, Hüllhorst, Germany), from 20 °C to 800 °C at a 10 °C min^−1^ heating rate under nitrogen flow. Thermal conductivity, *k*, was measured with a Thermal Constants Analyzer TPS 2500 S (Hot Disk, Göteborg, Sweden), using the transient plane source method with two samples maintained at 20 °C.

## 4. Conclusions

Samples with silica aerogel, rubber and PVB were successfully prepared using the hot-pressing technique.

The incorporation of recycled rubber into the samples did not lead to a negative impact on the final physical and thermal properties of the material, meaning that the final composites presented thermal conductivity (55.5 ± 0.5 mW m^−1^ K^−1^). The three composites showed excellent thermal stability with negligible weight loss up to 400 °C and losses lower than 8.5 wt% up to 600 °C. The addition of rubber also improved the mechanical properties; when compared with samples without rubber, the materials exhibited flexibility. The combination of these properties shows that the composites with aerogel and rubber here developed have the potential to be applied as a thermal insulation material in buildings. The use of small amounts of end-of-life tire rubber for this type of purpose can be a path to reduce it in the environment.

## Figures and Tables

**Table 1:** Static water contact angle of different samples.

Samples/Composites	Water Contact Angle (°)
P6	116.7 ± 2.4
P3A3	151.7 ± 4.6
P2A4	156.8 ± 3.1
P3R1A2	158.8 ± 2.1
P2R1A3	160.3 ± 3.6
P2R2A2	158.2 ± 4.8
R6	150.0 ± 4.5

**Table 2:** Mechanical properties of different samples and composites in single layer.

Samples and Composites	Young’s Modulus (kPa)	Compressive Stress after 10% Strain (kPa)	Recovery after 10% of Compression
P6	79.3	978	69
P3A3	75.4	587	84
P2A4	61.9	812	87
P3R1A2	12.9	855	84
P2R1A3	40.8	614	91
P2R2A2	93.2	652	90
R6	4.6	91	98

**Table 3:** Sample nomenclature.

Samples	Images	Composition
P6	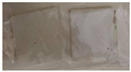	6 g PVB
P3A3	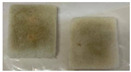	3 g PVB, 3 g Aerogel
P2A4	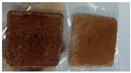	2 g PVB, 4 g Aerogel
P3R1A2	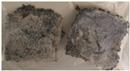	3 g PVB 1 g Rubber 2 g Aerogel
P2R1A3	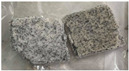	2 g PVB 1 g Rubber 3 g Aerogel
P2R2A2	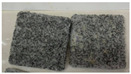	2 g PVB 2 g Rubber 2 g Aerogel
R6	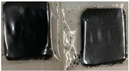	6 g Rubber

## Data Availability

Data are contained within the article. The raw/processed data required to reproduce these findings cannot be shared at this time due to technical or time limitations but will be sent upon request.

## Sample Availability

Samples of the compounds are available from the authors upon request.
